# Metagenome dataset of lateritic soil microbiota from Sadaipur, Birbhum, West Bengal, India

**DOI:** 10.1016/j.dib.2021.107041

**Published:** 2021-04-06

**Authors:** Meenakshi Mukhopadhyay, Arup Kumar Mitra, Sudeshna Shyam Choudhury, Sayak Ganguli

**Affiliations:** a Department of Botany, Vivekananda College, 269, Diamond Harbour Road, Kolkata, West Bengal 700063, India; b Post Graduate Department of Microbiology, St. Xavier's College (Autonomous), 30 Mother Teresa Sarani (Park Street), Kolkata, West Bengal 700016, India; c Post Graduate Department of Biotechnology, St. Xavier's College (Autonomous), 30 Mother Teresa Sarani (Park Street), Kolkata, West Bengal 700016, India

**Keywords:** Lateritic soil, Metagenomic profile, Illumina, Proteobacteria, Sustainable agriculture

## Abstract

The data represents the bacterial community profile obtained through metagenomic sequencing of soil sample, collected from the ‘Rarh’ region of West Bengal, which is characterized by the lateritic badlands dating back to the late Pleistocene. Taxonomic binning and operational taxonomic unit (OTU) prediction of the Illumina sequencing data indicated the abundance Proteobacteria (61%) followed closely by Bacterioidetes (35%). The top two most abundant genera identified, were Sphingobacterium and Acinetobacter respectively. Chemical properties of soil, such as pH, organic carbon content, available nitrogen, phosphorus, and potassium were also analyzed for enabling future researchers to correlate the abundance of microbial taxa with the prevalent conditions. These findings can be effectively used to formulate strategic microbiome engineering through bioaugmentation for a sustainable agricultural system.

## Specifications Table

**Table:** 

Subject	Microbiology
Specific subject area	Applied Microbiology and Biotechnology
Type of data	Raw Sequence Reads
How data were acquired	Shotgun metagenome sequencing and analysis
Data format	FASTQ file
Parameters for data collection	Environmental sample; lateritic soil with low nutrient content, susceptable to soil erosion, abandoned for two years after long-term cultivation
Description of data collection	Whole bacterial DNA extraction from soil using the PowerSoil® DNA isolation kit. The Illumina platform was used for Shotgun metagenomic sequencing.
Data source location	Latitude and longitude for collected samples/data: Sadaipur, Dubrajpur Block, Birbhum, West Bengal, India, located at 23.3260 °N, 87.4617 °E.
Data accessibility	Repository name: NCBI SRA
	Data identification number: PRJNA689214
	Accession No. SAMN17271210,
	Raw Sequence Reads_SAM1 Soil metagenome 410658.
	BioProject ID: PRJNA689214
	Direct URL to data: https://www.ncbi.nlm.nih.gov/bioproject/PRJNA689214

## Value of the Data

• The presented dataset is the first report on the status of microbiome in lateritic soil of ‘Rarh region’ of West Bengal. The soil is low in mineral nutrients, organic carbon content and has very low water holding capacity making agricultural practices difficult. Agricultural and environmental biologists will be benefitted from the present data.
• These data will help researchers to mitigate the problem of poor fertility by exploration of different microbial consortia and organic additives incorporated in traditional agricultural methods.
• From this data set, it may be possible to find out potentially beneficial soil bacteria having novel genes coding for enzymes with nutrient enhancing ability. A proper experiment can be designed based on these findings for utilization of these microbes to improve soil productivity for a sustainable agriculture.

## 1 Data Description

**Fig. 1:**
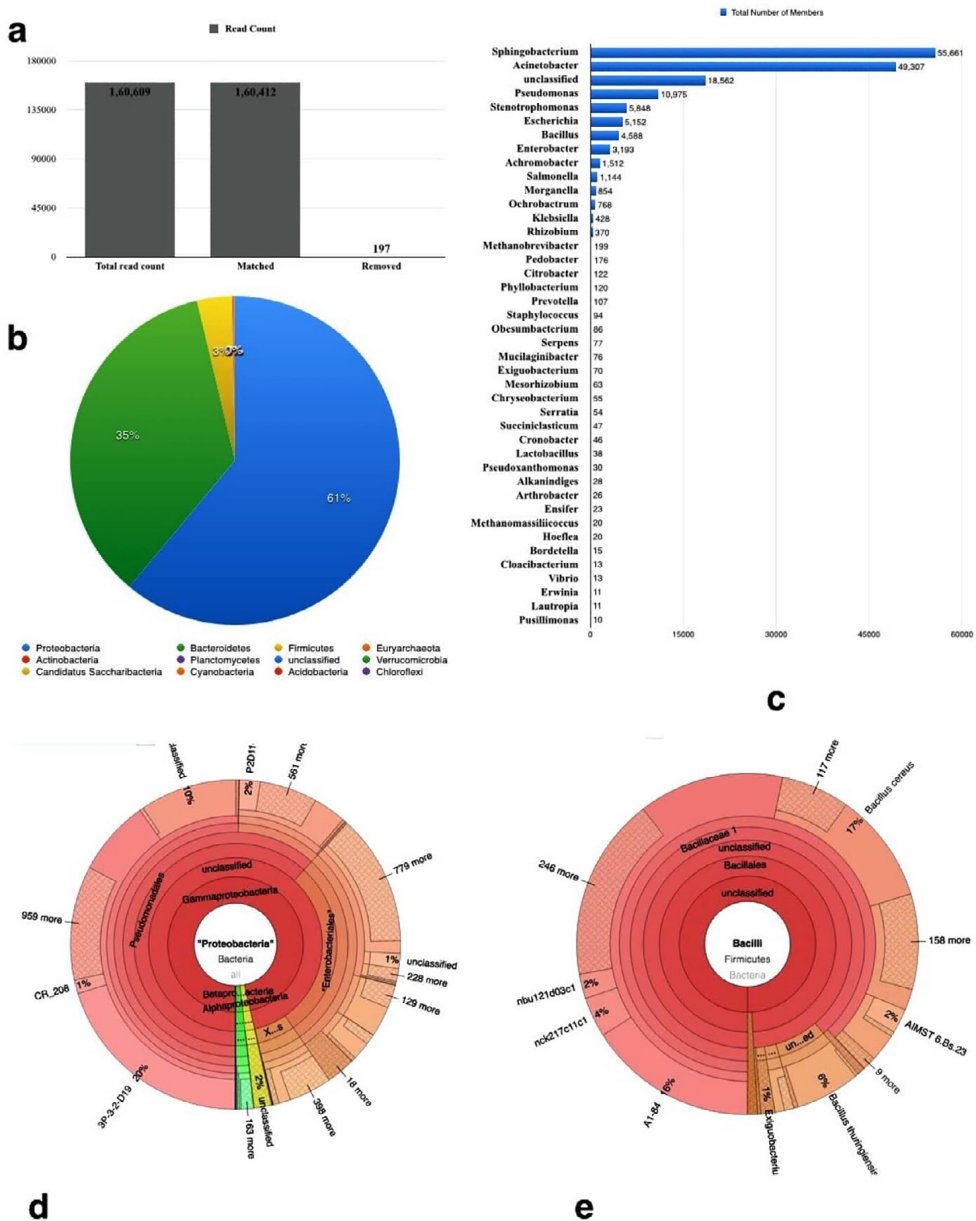
Data Profile obtained through sequencing. (a) Read Counts as obtained after initial quality check where 197 reads were discarded; (b) Phylum abundances indicating Proteobacterial abundance; (c) Genus level abundances which exhibits the predominance of *Sphingobacterium* and *Acinetobacter* (d) Phylogenetic representation using Krona chart to depict the distribution of Proteobacterial members; (e) Phylogenetic representation using Krona chart to depict the distribution of the *Bacillus* clade at the genus level.

Loose, friable, nutrient-depleted lateritic soil [1] was collected, and analyzed for the chemical properties like pH, organic carbon content, available nitrogen, phosphorus and potassium. (Table 1). The same soil was used for sequencing using Illumina Miseq platform, and a total of 1,60,609 reads were obtained, out of which 197 reads did not pass quality filtering step. Finally 1,60,412 reads were subsequently used for analysis (Fig. 1a). Only 2% of the reads represented Archaeal members and rest 98% were from Bacterial phylum. Proteobacterial abundance was predominant (Fig. 1b). At the genus level, Sphingobacterium (35%), Acinetobacter (31%) and Pseudomonas (7%) were the top three members in the total distribution of genera in the sample (Fig. 1c). Among the Proteobacterial members, Gammaproteobacteria (59%) was the most abundant followed by alpha, and beta, with predominance of Sphingobacteriaceae, Moraxallecaeae, Enterobacteriaceae, and Pseudomonadiales (Fig. 1d). In the Bacillus clade*, Bacillus cereus* (17%) and *Bacillus thuringensis* (6%) were identified to be the most abundant members (Fig. 1e).

**Table 1:** Chemical characterization of soil sample.

Soil Parameter	Result	Unit
pH at 25 °C	5.99	–
Total organic carbon	0.57± 0.011	%
Available Nitrogen (N)	81.91 ± 0.015	mg/kg
Available Phosphorus (P)	6.90 ± 0.01	mg/kg
Available Potassium (K)	126.57± 0.021	mg/kg

## 2 Experimental Design, Materials and Methods

### 2.1 Soil collection and chemical analysis

Soil sample was collected using the protocol as recommended by TNAU-2013 [2]. Chemical analysis was performed to estimate the pH, organic carbon content, available nitrogen, potassium, and phosphorus using the standard methods as followed in [3].

### 2.2 Next generation sequencing and metagenomic analysis

Metagenomic DNA extraction from the sieved soils was carried out using the PowerSoil™ DNA isolation kit (MOBIO), following which the sample was sequenced using the manufacturer's protocol. DNA quality was analyzed by Nanodrop and then evaluated on agarose gel. It was quantified using QUBIT. The library preparation was carried out using Illumina standardized V3-V4 regions of the 16S rRNA gene library protocol. The enriched library was further quantified and validated using qPCR and Agilent Bioanalyzer (DNA 1000 chip). The library generated containing V3-V4 amplicons [Primer Details: 16S Amplicon PCR Forward Primer = 5′TCGTCGGCAGCGTCAGATGTGTATAAGAGACAGCCTACGGGNGGCWGCAG;16S Amplicon PCR Reverse Primer = 5′GTCTCGTGGGCTCGGAGATGTGTATAAGAGACAGGACTACHVGGGTATCTAATCC and adaptor sequences: Forward overhang: 5′ TCGTCGGCAGCGTCAGATGTGTATAAGAGACAG (locus specific sequence) Reverse overhang: 5′ GTCTCGTGGGCTCGGAGATGTGTATAAGAGACAG(locus specific sequence) 341F = CCTACGGGNGGCWGCAG and 805R = GACTACHVGGGTATCTAATCC] was sequenced on Illumina MiSeq using reagent kit V3 for generating 2 × 300 bp read length. The sequenced raw data files were passed through Quality check using the FASTQC pipeline, and sequences which passed the quality screening were then used for assembly using the SILVSngs (1.3) platform. It involved homopolymer removal [4] along with discarding of artifacts and contaminations [5]. Qiime was used to cluster the operational taxonomic units (OTUs), and KRONA charts were generated to analyze the microbial abundances. The pipeline followed was in accordance with the methods described in [6] and [7].

## Funding

This work was financially supported by the Department of Higher Education, Science Technology and Biotechnology, Govt. of West Bengal, India [Memo No. 528 (Sanc.)/ST/P/S&T/2G-31/2018 Dated 26.03.2019]

## Ethics Statement

Not applicable.

## CRediT Author Statement

**Meenakshi Mukhopadhyay:** Conceptualization, Investigation, Data curation, Funding acquisition; Writing - Original Draft, Writing - Review & Editing; Formal Analysis; **Arup Kumar Mitra:** Conceptualization, Supervision; **Sudeshna Shyam Choudhury:** Supervision; **Sayak Ganguli:** Writing, Reviewing, Editing.

## Declaration of Competing Interest

The authors declare that they have no known competing financial interests or personal relationships that could have appeared to influence the work reported in this paper.
